# A road map for designing and implementing a biological monitoring program

**DOI:** 10.1007/s10661-016-5397-x

**Published:** 2016-06-08

**Authors:** Joel H. Reynolds, Melinda G. Knutson, Ken B. Newman, Emily D. Silverman, William L. Thompson

**Affiliations:** Western Alaska Landscape Conservation Cooperative, Anchorage, AK 99503 USA; National Wildlife Refuge System, US Fish and Wildlife Service, La Crosse, WI 54603 USA; Lodi Fish and Wildlife Office, US Fish and Wildlife Service, Lodi, CA 95240 USA; Division of Migratory Bird Management, US Fish and Wildlife Service, Laurel, MD 20708-4002 USA; National Wildlife Refuge System, US Fish and Wildlife Service, Hadley, MA 01035 USA

**Keywords:** Monitoring design, Effectiveness monitoring, Status and trends monitoring, Adaptive management, Inventory, Structured decision making

## Abstract

Designing and implementing natural resource monitoring is a challenging endeavor undertaken by many agencies, NGOs, and citizen groups worldwide. Yet many monitoring programs fail to deliver useful information for a variety of administrative (staffing, documentation, and funding) or technical (sampling design and data analysis) reasons. Programs risk failure if they lack a clear motivating problem or question, explicit objectives linked to this problem or question, and a comprehensive conceptual model of the system under study. Designers must consider what “success” looks like from a resource management perspective, how desired outcomes translate to appropriate attributes to monitor, and how they will be measured. All such efforts should be filtered through the question “Why is this important?” Failing to address these considerations will produce a program that fails to deliver the desired information. We addressed these issues through creation of a “road map” for designing and implementing a monitoring program, synthesizing multiple aspects of a monitoring program into a single, overarching framework. The road map emphasizes linkages among core decisions to ensure alignment of all components, from problem framing through technical details of data collection and analysis, to program administration. Following this framework will help avoid common pitfalls, keep projects on track and budgets realistic, and aid in program evaluations. The road map has proved useful for monitoring by individuals and teams, those planning new monitoring, and those reviewing existing monitoring and for staff with a wide range of technical and scientific skills.

## Introduction

Long-term monitoring of natural resources is of growing importance (Janetos and Kenney 2015), especially at larger spatial scales (e.g., Beever and Woodward 2011; Christensen et al. 2013; Gannon et al. 2013; Kenney and Janetos 2014; Isaak et al. 2015; Janetos and Kenney 2015). Yet, despite the growing demand for data collected consistently across space and time, too often, monitoring efforts fail (Field et al. 2007; Reynolds 2012). The literature is rich with monitoring guidance and lessons learned that highlight the diverse sources of failure—from poorly defined objectives (e.g., Silsbee and Peterson 1993; Lindenmayer and Likens 2010b), poor selection of indicators (Hinds 1984; Olsen et al. 1999; Irvine et al. 2015), inadequate survey design or statistical power/survey effort (Taylor et al. 2007; Reynolds 2012), or the more complex organizational issues that arise in sustaining program consistency across timescales of a decade or more (Lindenmayer and Likens 2010a). The literature broadly clusters into two groups—those focused on general issues of problem framing and objective setting and those focused on detailed guidance regarding specific technical issues (e.g., indicator selection, survey design, and analysis). Although well aimed and informative, neither group covers all the requisite components required for a successful monitoring program, let alone how the decisions in any one component influence and/or constrain the choices in any other component (Reynolds 2012). This leaves those charged with developing a new monitoring program, or sustaining an existing one, to develop their own holistic vision of these many components and linkages—a task requiring time and usually achieved in hindsight.

When initiating a new monitoring program, the lack of an overarching vision of all the necessary components presents two fundamental barriers to success. First, it hinders recognition of the diversity of considerations and decisions required and, thus, recognition of both the preliminary planning necessary prior to actual data collection and the resources necessary for success. Second, given that monitoring efforts generally require high-functioning collaborative teams, the lack of a high-level organizational structure hinders the clear and effective communication required to ensure that all team members are aligned on the same goal and aware of how their specific contribution (e.g., coordination, logistics, statistical design and analysis, data management, communications) links with others (National Research Council 2015). Indeed, the biggest challenges that undermine the success of monitoring programs are cultural and organizational (Field et al. 2007). Successful monitoring requires strong collaboration among managers, ecologists, and data scientists that is hindered by organizational boundaries, funding cycles that are too short, and the common practice of allowing data to pile up without rigorous analyses that would inform improvements in monitoring methods.

**Fig. 1:**
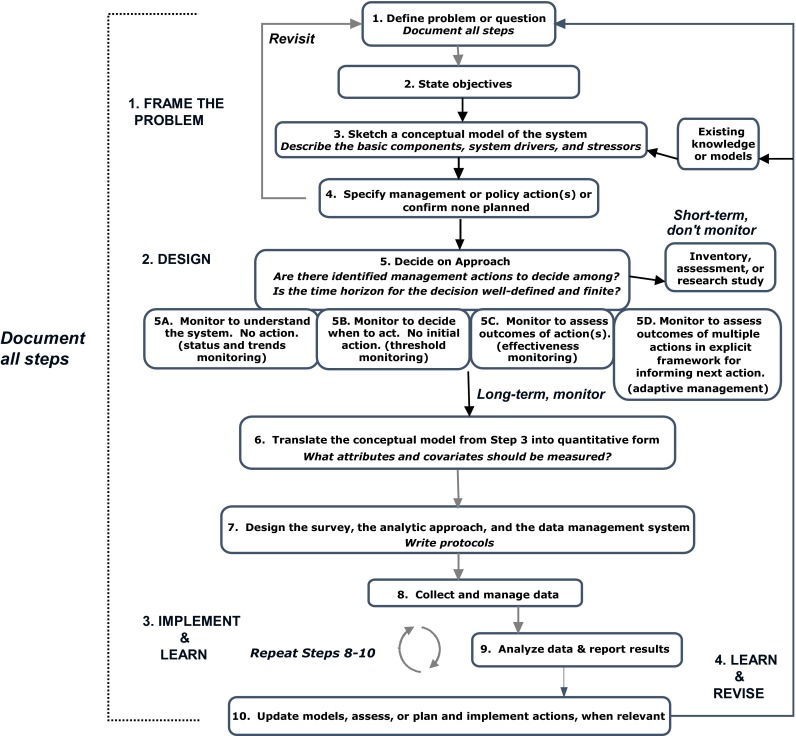
The road map for designing and implementing monitoring has ten steps sequenced in four general phases—“frame the problem,” “design,” “implement and learn,” and “learn and revise.” Key times for iteration back through earlier steps are denoted by the *return arrows* emerging from step 4, step 10, and phase 4 *learn and revise*. Decisions made at each step should be recorded in the project record (Appendix)

We have addressed these challenges by creating a ten-step “road map” for designing and implementing a biological monitoring program (Fig. 1). The road map provides a high-level overview of the full monitoring process: from framing the problem and defining objectives, to deciding if monitoring is even required, to the technical details of survey design and data management, to the administrative elements required to ensure program learning and improvement. In addition to providing for more effective communication, collaboration, and team alignment, the graphical synthesis can be used for benchmarking to ensure that projects remain on track and resources are scheduled and available.

The road map can be seen as an extension of earlier graphical efforts (Hinds 1984; Silsbee and Peterson 1993; Elzinga et al. 2001; Lindenmayer and Likens 2009; Lindenmayer and Likens 2010a) and a synthesis of earlier design principles, both general (Silsbee and Peterson 1993; Wissmar 1993; Maher et al. 1994; Elzinga et al. 2001; Lindenmayer and Likens 2010b; Lindenmayer and Likens 2010a) and technical (Olsen et al. 1999; McDonald 2003; Taylor et al. 2007; Reynolds 2012). It is more comprehensive and prescriptive than these earlier efforts in emphasizing linkages among the myriad planning decisions, as well as, in the accompanying narrative, providing guidance on specific methods, tools, or resources that we have found to be most effective for each step. The road map’s philosophy is aligned with the adaptive monitoring approach espoused by Lindenmayer and Likens (2009) though it is somewhat broader in the inclusion of specific guidance for deciding if monitoring is even required (step 5) and, if so, what type of monitoring.

In 1993, this journal devoted a special issue to papers from a workshop on “Improving Natural Resource Management through Monitoring.” The opening editorial commented that “(a)lthough most of the concepts may seem self-evident, they are widely applicable and should be explicitly incorporated into the planning and implementation of any…monitoring program” (Stafford 1993) (pg. 87). Almost 25 years on, the literature demonstrates that when it comes to the complex challenges of long-term natural resource monitoring, these “self-evident” concepts continue to be so mainly in hindsight and are still not widely recognized and adopted. Our objective in creating the road map is to help change that situation over the next 25 years.

## A road map for designing and implementing a biological monitoring program

The ten-step road map for designing and implementing a monitoring program has four general phases (Fig. 1):

1. *Frame the problem*, clarify the objectives, develop a conceptual model of the system, and identify possible management actions
2. *Design* the monitoring, including the data collection, analysis, and data management components. This entails first deciding whether or not monitoring is even required
3. *Implement* the monitoring *and learn* from the data; inform decision making
4. *Learn* to improve the monitoring process; incorporate new tools and system information; *and revise* objectives, design, and methods, as appropriate

We review each phase of the road map, briefly describing the core goals, actions, and products. We use a simple example of sustaining a moose population to illustrate the steps. The more technical aspects of monitoring, by necessity, are described only superficially; our focus is providing guidance on overall program planning and design to maximize value in support of policy or management decision making.

### Phase 1—frame the problem

Steps 1–4 of the road map (Fig. 1) follow a structured decision making process (Lyons et al. 2008; Gregory et al. 2012) and include key tools useful in decomposing a problem to determine the appropriate approach.

#### Step 1: define the problem or question

A management problem or question motivates the need for information about the system. A robust problem definition should answer “Why is this an important problem or question?” and address the following: the temporal and geographic scope of the problem; who has the authority to make decisions that could resolve or address the problem; the legal, financial, or political constraints that the decision makers are working under; what information about the resource is needed to improve the decision making; and who are the stakeholders that will be interested in and/or impacted by the decision. It is not uncommon for monitoring data to have proximate value to the planning team and stakeholders, as well as long-term value to future decision or policy makers and the conservation science community. A good understanding of multiple information needs and pathways to influence decision making should be considered in this step. This “information need” is what the monitoring effort aims to address (Reynolds 2012).

In resource management agencies, it is often difficult to discern exactly what the problem is, especially if several people or stakeholders need to arrive at a common understanding. Indeed, problem definition is often skipped in favor of immediately focusing on what to monitor. However, problem definition is required to place monitoring within the relevant management context. Even when the focus is simply the “status and trend” of a resource, there is an unstated expectation that someone will endeavor to achieve or maintain a desirable state or reverse an undesirable trend. Who has that responsibility and authority? This person, office, or agency will be reading the reports and using the information to guide decisions. They need to be involved in the planning, so they can clarify their information needs, including specifying the desired precision and quality of information, the timing and format of reporting, and better understand what information is feasible to obtain (Averill et al. 2010). A workshop provides a positive setting for the focused, cross-disciplinary dialogues usually required to clarify these information needs. This is the foundational step of the monitoring design process because it clarifies the underlying goal of the monitoring; failing to specify the problem is like building a house on a weak foundation.

#### Step 2: state the objectives

Once the problem is defined, one needs to articulate the objectives. Objectives often relate to a desired future condition of a resource, although they sometimes entail simply understanding the current condition (Keeney 1992; Yoccoz et al. 2001). In the former case, a manager may want to restore ecological integrity to a degraded system or ensure certain levels of ecosystem services.

**Fig. 2:**
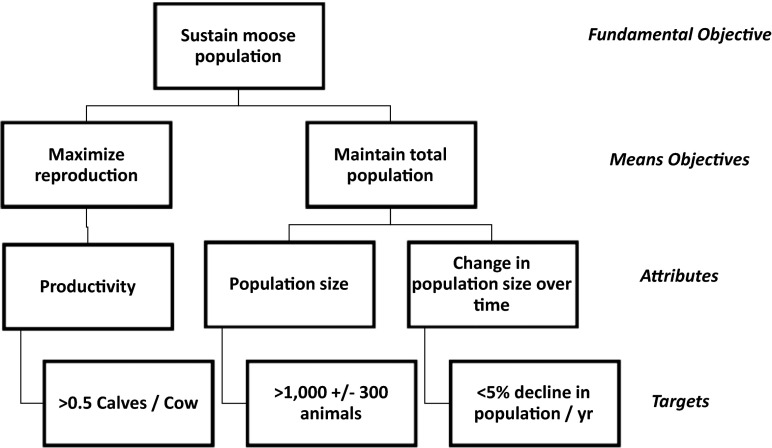
The objective hierarchy for the moose example, illustrating the relationships among the fundamental objective (“Sustain moose population”), means objectives (e.g., “Maximize reproduction”), attributes (i.e., the characteristics of the system that are of interest, such as “Productivity,” see “Step 6: translate the conceptual model from step 3 into quantitative form” section), and targets and thresholds based on the measurements selected for each attribute (e.g., “>0.5 calves/cow”). The objective hierarchy is developed and refined during steps 2 and 6. The moose example involves annual decisions and, based on the objective hierarchy, requires annual surveys in each of the five management units to estimate productivity attributes of the moose population in each unit and overall population size and trends

There are two types of objectives: fundamental and means. Fundamental objectives are the core outcomes that one cares about “just because” and represent something to strive for to achieve the organization’s mission. A good fundamental objective is the “broadest objective that will be directly influenced by the [decision] alternatives and within the control of the decision maker” (Gregory et al. 2012, p. 74). In natural resources management, fundamental objectives usually represent a healthy ecosystem, habitat, or population. Means objectives contribute to achievement of the fundamental objectives by defining a particular way of achieving the fundamental objective. For example, given a fundamental objective of “prevent an endangered bird species from going extinct,” an associated means objective might be to “increase reproductive success.” The relationships between the fundamental and means objectives can be depicted graphically by an objective hierarchy; Fig. 2 illustrates an objective hierarchy for a fundamental objective of sustaining a moose population.

Step 2 involves clarifying the fundamental and means objectives and sketching the objective hierarchy. In step 6 (Fig. 1), we will return to the objective hierarchy to step down each means objective into system characteristics (“attributes”) that can be measured to assess achievement of the means objective—these are already shown in Fig. 2.

#### Step 3: sketch a conceptual model of the system

Given an identified problem and objectives, developing a conceptual model (step 3) provides an essential perspective (Fancy et al. 2009; Margoluis et al. 2009) by showing key system components, including threats (Salafsky et al. 2008), human activities or interventions, and their relationships to the fundamental objective. The *conceptual model of the system is the intellectual foundation upon which a monitoring program rests*, as it makes explicit the connection between system drivers (including management actions) and the fundamental objectives, thus helping clarify exactly what should be monitored (Ogden et al. 2005; Woodward and Beever 2011). Existing information about the system informs the conceptual model; current knowledge of the literature is essential for building a useful conceptual model.

**Fig. 3:**
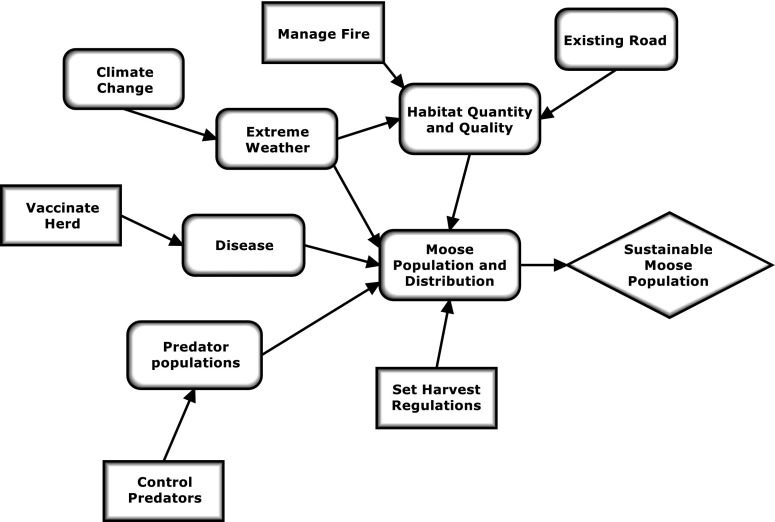
The influence diagram, developed in step 3, is a conceptual model that illustrates the “big picture” associated with a problem and indicates where decisions or actions could be applied and their assumed pathways of influence. This diagram depicts factors and actions that can potentially affect the fundamental objective (*diamond*) of sustaining the moose population in the example. Contributing factors (*rounded rectangles*) include things that may respond to management actions (habitat quantity and quality, disease, predator populations) as well as factors that are largely outside the manager’s control (extreme weather). *Rectangles* denote potential management actions

Figure 3 illustrates one type of conceptual model, an influence diagram, for the moose population example. Geometric shapes visually distinguish the fundamental objectives, important system components or drivers, and management actions and show the connections between each. The initial version of the influence diagram is often complex and, with iteration, will be refined and simplified. An influence diagram can provide insights about how the system functions and should include drivers impacting the fundamental objective, possible management actions (including policy decisions) that could reduce the negative impacts of drivers (“threats”) and/or promote achievement of the fundamental objective, and external factors that need to be accounted for in design and analysis of monitoring (Margoluis et al. 2009; Gregory et al. 2012). For example, predator control used to manage moose populations is a controversial management action. The influence diagram should include both the intervening external factors that may moderate the impact of predator control as well as other possible management actions (harvest regulations, habitat enhancement) that may similarly increase moose populations, thus providing other management alternatives. Ideally, the influence diagram becomes the basis for future process or quantitative models (step 6) (Conroy and Peterson 2013). Utility nodes can be added to the influence diagram to incorporate explicitly the “value,” positive or negative, associated with different actions and outcomes (Conroy and Peterson 2013).

Conceptual models are also useful for clarifying the four main types of uncertainties associated with management decision making (Nichols et al. 2011). These uncertainties are as follows: *environmental variation*—inherent stochastic variation in environmental factors; *partial controllability*—imprecise application of management actions; *partial observability*—imprecise measurement of the system; and *structural uncertainty*—lack of understanding about relationships between system state and drivers, e.g., uncertainty in defining the model itself. Knowing the major uncertainties that adversely affect decision making allows one to design the monitoring program to control and/or reduce each of them, as feasible (Yoccoz et al. 2001; Regan et al. 2002; Kendall and Moore 2012). Referring to Fig. 3, extreme weather is associated with environmental variation; partial controllability may be associated with both predator control and vaccination (impossible to vaccinate all animals or control certain wolf packs) and should be considered in defining any related actions. Partial observability will be a factor in all observations made of the system and should be accounted for in the design and measurement process. Structural uncertainty can be addressed through careful design of the monitoring approach (step 5) if its reduction is identified as a means objective.

#### Step 4: specify management or policy actions or confirm none planned

Are the decision makers and stakeholders considering potentially implementing specific management or policy actions to resolve or address the problem? To avoid confusion, we reserve the term “action” to mean management activities intended to relatively directly reduce a threat or otherwise improve the state of the system under management; we do not consider as an action “implementing a procedure to inform or assess the effects of management actions, e.g., starting a monitoring program.” Examples of actions include habitat manipulations, regulations and restrictions, policies, funding decisions (including funding research to reduce structural uncertainty regarding how the system will respond to specific actions), administrative adjustments, and “doing nothing.” Brainstorming a wide range of potential actions with limited censorship will encourage novel ideas to emerge (De Bono 1989), only then begin filtering the suite of potential actions by considering logistics, legality, political palatability, resources, and time. If there are no potential actions being considered, go to step 5; otherwise, continue in step 4 clarifying the actions.

For each action remaining under consideration, use the conceptual model from step 3 to develop a “results chain” (Margoluis et al. 2013)—a path through the influence diagram specifying a sequence of cause-and-effect relationships that begin with the action, possibly run through a series of intermediate impacts, and end with a means objective that the action is expected to impact. The results chain summarizes how the system is expected to change if the action is implemented and thus encapsulates your “theory of change” (Margoluis et al. 2013) For the moose population example, one results chain is as follows: Vaccinate herd for brucellosis in fall → decrease incidence of disease → increase male overwinter survival and maternal health → improve productivity per female → increase net productivity of population. By clarifying the path of influence for a specific action, a results chain may suggest additional, intermediate system characteristics worth measuring to track effectiveness and identify where your system understanding may be breaking down (step 6), perhaps due to structural uncertainty; it may reveal intervening factors that need to be considered or perhaps even controlled, and it can aid in the development of quantitative models describing system dynamics (step 6). At the end of this process, update the conceptual model if necessary.

In clarifying the details of each action, including the associated results chain, be sure to specify operational details such as the following: (i) Is it a one-time action or will it be repeated? If repeated, how often? (ii) When should the action be applied? (iii) Are multiple actions possible at a given point in time? (iv) How quickly is the system is expected to respond to the action? Is there likely to be a long lag time? (v) How much control is there over implementing the action? Is there large uncertainty (e.g., partial controllability)? (vi) How much will it cost to implement the action?

Often, management actions can be grouped together into portfolios (Doremus 2003; Blomquist et al. 2010), e.g., sets of actions that can be implemented together. The conceptual model for the moose population (Fig. 3) shows four types of actions: control predators, set harvest regulations, vaccinate the herd, and manage fire. These can be combined, mixing different implementation options for each action type into different portfolios, e.g., varying harvest season length and bag limits.

#### Revisit steps 1–4 and create administrative record

Moving quickly and repeatedly through steps 1–4 (“rapid prototyping”) is an effective and efficient way to “frame the problem” (Starfield et al. 1994; Blomquist et al. 2010). In practice, each step in the process informs both earlier and subsequent steps, leading to refinement of the problem, objectives, alternatives, and, eventually, the entire conceptual model of the system. The “revisit” arrow (Fig. 1) is a reminder to reconsider the initial steps before proceeding to step 5; this might be done multiple times.

When one is satisfied that steps 1–4 have been adequately completed, at least for the time being, one should create an administrative record for the project (project record, Appendix). This documents the project’s history and evolution, recording decisions made during each step of the road map. Details of both decisions and their rationale are easily forgotten if not documented. This can cause decisions to be revisited repeatedly and needlessly, impeding progress (and engagement). It may take many months, perhaps even a year or more, to fully design a monitoring program; during the interim, people could forget how or why they made certain decisions.

The project record provides a condensed summary of key information from project documents such as meeting notes, workshops, conference call minutes, protocols, survey designs, fact sheets, lists of participants, survey data, reports, etc. It is important to record modifications to the survey design, protocols, or management actions. To avoid bias or confounding, these modifications must be accounted for, not only in the data analysis, but perhaps also in future data collections. Staff changes, even at the level of survey coordinators or principal investigators, are inevitable in monitoring programs that span multiple years. Clear and thorough documentation is essential if a monitoring program is to survive staff turnover.

### Phase 2—design

Once the decision context has been clearly defined and the associated information needs have been identified, one must decide on the best approach for obtaining the information (step 5), in particular, *whether monitoring is even necessary*. If monitoring is deemed necessary, then one proceeds to develop the technical details for the data collection, analyses, and data management (steps 6 and 7). The design phase involves a number of technical decisions that will ultimately determine the effectiveness and efficiency of the monitoring. These steps should be undertaken in consultation with experts in statistics and data management.

#### Step 5: decide on the approach

After completing steps 1 through 4, one has a clear understanding of the motivating problem and is ready to determine the most efficient way to obtain the needed information. New data collection may not be necessary; the required information may be available in the published literature or from analysis of existing data sets. Using existing information is faster and more cost-effective than monitoring. Because the published literature may contain information that has escaped the attention of the decision makers, scientists on the team may resolve the problem by identifying and summarizing the relevant literature. In the moose example (Fig. 3), there may be research demonstrating the effectiveness, or not, of either vaccination or predator control in controlling moose populations. The possibility that the needed information is already known provides motivation to review the available literature and build relationships with experts who have this knowledge. Among a manager’s many responsibilities is the professional responsibility to help develop best management practices by summarizing and sharing their own experiences and data in the appropriate published literature, thus helping others in similar situations. To facilitate such communications, time and money for producing these summaries must be included in project proposals. Simplifying the sharing of conservation management lessons in order to improve learning by the conservation community is a major goal of recent efforts to develop standardized vocabularies (e.g., Salafsky et al. 2008) and libraries of common management decision frameworks (e.g., Muir et al. 2014 and the Conservation Actions and Measures library at www.miradishare.org).

If the needed information is unavailable, it is prudent to next ask “is it worth gathering?” before undertaking a new investigation. Costs, including time, may be a significant factor in decisions about what approach should be taken. Indeed, a manager must decide whether the optimal decision is to invest in management rather than monitoring (Field et al. 2004). Major considerations include the degree of uncertainty regarding how the system will respond to the action (due to each of the types of uncertainties mentioned earlier, especially environmental and structural) and the risks associated with delayed or no action. A rough tally of monitoring costs can be developed by estimating the costs with each of the remaining steps in the road map (Fig. 1). Formal methods of examining tradeoffs in cost and effort involve expanding the influence diagram to include utility nodes (Conroy and Peterson 2013) and using the resulting decision model to estimate the value of information that will be gained via monitoring compared with other information-generating approaches or with making decisions in the absence of the desired information (Runge et al. 2011).

**Fig. 4:**
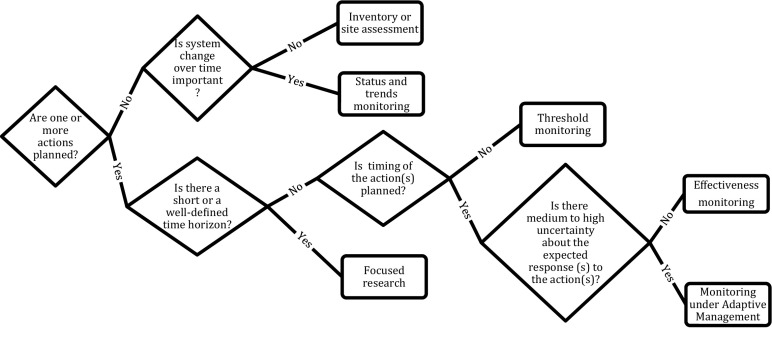
The type of monitoring required (step 5) can be decided by clarifying whether there are any specific management actions under consideration, the amount of time available for making decisions regarding actions (“time horizon”), the timing of planned actions, and the relative magnitude of uncertainty regarding how the system will respond to the action(s). When appropriate and feasible, focused research is likely to be faster and less costly than monitoring. Adaptive management is only possible when there are iterative decisions, e.g., repeated decisions of the same kind are made over time or across space (see text of “Step 5: decide on the approach” section for further details)

If the relevant information is unavailable but deemed worth acquiring, then one needs to decide how to collect or generate the information. The decision tree shown in Fig. 4 can help with sorting through the variety of approaches—research, inventory, or monitoring—based on the types of information needed, whether a management action is planned, the timing of the action(s), the level of uncertainty associated with the expected response(s) to the action(s), and the time frame available for collecting the needed information.

##### Research

Focused research to determine the best management action may be appropriate if there are one or more actions under consideration (e.g., vaccinating moose), structural uncertainty is the main concern, and the system is expected to respond to manipulations within the time period available for managers to decide which action to implement *at actual scale* (e.g., across a whole conservation unit)—in Fig. 4, this is summarized as having a “well-defined time horizon” (especially a short one). For example, if managers are deciding among competing herbicide treatments to adopt for use in reducing an invasive plant on a relatively large conservation unit, they may be able to conduct research, perhaps on a small portion of the unit, for a season to assess their effectiveness prior to deciding on which herbicide to implement more broadly. This approach is most effective for frameworks where there is high controllability and low to moderate environmental uncertainty.

In some cases, the effects of two or more actions can be evaluated using designed experiments, which can establish a causal relationship between an action and an outcome. Well-designed experiments have two key features: (i) treatments (e.g., actions) must be randomly assigned to experimental units (plots, animals, trees, etc.) and (ii) each treatment must be applied independently to at least two units (Cox and Reid 2000). Designed experiments accelerate learning by controlling for extraneous sources of variation in a systematic way. If the problem will yield to this approach, it is likely quicker and more cost-effective than monitoring. Many questions involving animal behavior or contaminants can be answered with a series of designed experiments, such as determining safe vaccine dosages for the moose example introduced earlier.

Alas, designed experiments are often infeasible in natural resource contexts. Treatments may not be repeatable (e.g., removal of a large dam), or their spatial-temporal extent may make application to multiple experimental units infeasible (e.g., a state-wide change in moose harvest regulations). Often, there is inadequate ability to consistently apply the treatment (e.g., partial controllability), such as the imposition of size limits for trout in a recreational fishery, or consistently assess the outcome (e.g., partial observability), such as often holds for actions targeted at affecting human behavior via policy, enforcement or education (e.g., implementing an education program to reduce the spread of invasive aquatic species). Adaptive management approaches (described below) were largely developed to overcome these challenges, allowing knowledge of treatment options gained through limited-scale designed experiments to be tested in the field under “real” conditions. For example, treatment options for invasive species are often tested first in a series of greenhouse experiments, followed by field trials, and finally under real management conditions.

##### Inventory

A one-time inventory or site assessment is appropriate when there is no management action under consideration and when the objectives can be met over a relatively short time interval (for example, change over time is not important). Referring to the moose management example (Fig. 3), if little is known about the spatial distribution, abundance, or age and sex structure of a population, then an initial inventory of the population is necessary.

##### Types of monitoring

Once a decision has been made to pursue monitoring, it is necessary to identify the most appropriate form of monitoring. Monitoring efforts have been classified by a diversity of criteria and terminology: for example, passive/mandated/question-driven sensu Lindenmayer and Likens (2010a) and targeted/cause-and-effect/context sensu Rowland and Vojta (2013). For the purpose of guiding design decisions, we define four types of monitoring, differentiated by their relationship to management action(s):

- If no specific action is being considered and the purpose is simply characterization of the state of a system over time, e.g., how many moose are present in a unit over time, then *status and trends monitoring* is appropriate (Fig. 1, box 5A).
- If the monitoring information will trigger a specific action, e.g., burn a management unit, *threshold monitoring* is appropriate (Fig. 1, box 5B).
- If timing of an action is planned and there is relatively low uncertainty regarding the expected response to the action, e.g., a moose vaccine is generally known to be adequate in field conditions, then *effectiveness monitoring* is appropriate (Fig. 1, box 5C).
- If timing of an action is planned, there is medium-high uncertainty in the expected response to the action, and alternative actions will be formally compared to better inform future decisions, e.g., a goal is to reduce structural uncertainty as to relative effectiveness of burning versus other silvicultural treatments in a forest, then formal *monitoring in an adaptive management framework* is appropriate (Fig. 1, box 5D).

The four types of monitoring distinguish management information needs and contexts, and the intent to employ one or the other affects design decisions, as we describe in step 7. Although all four focus on uncertainty regarding the state of the system, two focus on system state without or before an action (status and trends and threshold monitoring) and two focus on the system’s response to action (effectiveness monitoring and adaptive management). Taken together, the number of actions (if any), the timing of the actions, and the degree of structural uncertainty (which influences the framework for informing future decision making) define the type of monitoring or study that should be designed (Fig. 4).

##### Status and trends

The defining characteristic of status and trends monitoring [sometimes called baseline, surveillance, or passive monitoring (Lindenmayer and Likens 2010a)] is its focus on the state of the system, independent of any management actions (Morton et al. 2008). The purpose is to estimate the status and trend of some component of the system (Fancy and Bennetts 2012), often because a threat to the system is anticipated and monitoring is required to establish current conditions (“baseline”), including natural ranges of variability, or to detect a change. For example, concerns over how climate change may affect the moose population might motivate monitoring to track population abundance or spatial distribution. Sometimes, legislation mandates monitoring [regulatory monitoring (Lindenmayer and Likens 2010a)]. Even in these contexts, a conceptual model of the system is essential to guide the selection of the appropriate measurements.

There are several problems with status and trends monitoring (Nichols and Williams 2006). First, there is typically no clear timeline or milestone to prompt assessment, revision, or termination of the monitoring. Second, maintaining the monitoring long-term is challenging when no one directly relies on the products of the monitoring for decision making. Focusing on system state independent of any management decisions can make it difficult to find committed champions who will support the program during times of lean budgets and staff reductions [though see the successful approach of the US Forest Service’s Forest Inventory and Health Assessment program (Woodall et al. 2011)]. Third, without a decision maker demanding information in a timely manner, data management, analysis, and reporting (steps 9–10) may be delayed or neglected. Problems with survey design can persist undetected and unresolved, wasting resources, and, potentially, failing to detect degradation of the natural resources being monitored (Reynolds et al. 2011). Fourth, the information from this type of monitoring can be difficult to interpret if the major drivers of change are not also measured. Long-term status and trend information may be of general interest but often lacks specificity in terms of providing guidance for action, since management was not incorporated into the survey design. For example, if a species has declined to a point where some management intervention is required, long-term baseline monitoring may indicate that urgent action is needed but not which action(s) will be most effective. Species declines simply trigger further study in the form of research to determine causes of the decline and possible treatments.

##### Threshold monitoring

If specific actions are anticipated, monitoring can be designed to efficiently inform the decision to act. The addition of a decision context might call for management response when the system state reaches a pre-defined threshold (Martin et al. 2009; Rogers et al. 2013). Threshold monitoring is often used for management of processes with known directionality, such as plant succession, timber stand improvement, or deterioration of roads or trails by erosion or high visitor use. For example, when the cover of woody species in a grassland unit reaches a specified level, a manager with the objective of maintaining the grassland will initiate some disturbance (fire, grazing, mowing) to set back succession and reduce the dominance of woody species. This type of monitoring is appropriate when the system response to the planned action is already well established and known with great certainty.

##### Effectiveness monitoring

Once the decision to act has been made, managers, funders, and/or other stakeholders should want to learn the action’s consequences (e.g., Margoluis et al. 2013). This applies even if structural uncertainty is low, especially when there is large environmental uncertainty influencing the system response. In its most basic form, effectiveness monitoring involves documenting system response and noting the degree to which the desired outcome was attained. For example, effectiveness monitoring might be conducted to assess the effect of a change in harvest regulations on moose population size. Note that the term “effectiveness monitoring” occurs in a variety of contexts besides resource management and conservation delivery; regardless of context, the intent is to assess how well a desired outcome was attained.

The resulting information can be used to trigger continued action (e.g., “Was the density of trees reduced enough or is more action required?”) and improve prediction of system response to future actions (Parrish et al. 2003). However, if the focus is predominantly on reducing such structural uncertainty, the information needs may be resolved more quickly via literature review or research (Fig. 4). If enough documented reports of outcomes from previous actions are available, a meta-analysis may be possible to help identify best management practices (Roscoe and Hinch 2010).

When there is medium to high uncertainty regarding how the system will respond to the actions (e.g., predator control), effectiveness monitoring may not be very informative if monitoring is restricted to just the targeted means objectives (e.g., moose abundance) (Allen et al. 2011). In such cases, if the desired outcome is not attained, subsequent actions are reactive and often ad hoc, requiring a return to phase 1 (frame the problem) to identify alternative strategies and competing system models. This is avoided by spending adequate time in step 4, *Specify management or policy actions or confirm none planned*, thoroughly developing the results chain associated with each potential action and identifying appropriate intermediate system characteristics to monitor (e.g., moose calf and female survival), in addition to the targeted means objectives, to identify any breakdowns in the conceptual model of the system (see Margoluis et al. 2013).

##### Adaptive management

Adaptive management is a formal framework for iterative decision making in the face of large uncertainty regarding how a system will respond to a set of potential actions. It uses a quantitative “learning process” to combine (i) effectiveness monitoring and (ii) a suite of predictive models quantifying the expected outcomes from each potential action (see step 6) to distinguish actions that move the system in the desired direction from those that have no or negative effect (Allen et al. 2011; Williams 2011). The approach can generate timely information about which management options work under which conditions (Knutson et al. 2010; Gannon et al. 2013), providing guidance on the optimal action given current system state. While more frequent replication of management actions (in space or time) leads to faster reduction of structural uncertainty regarding system response (Williams et al. 2009), the rate of learning also strongly depends on the system response time and environmental uncertainty. Adaptive management uses monitoring both to reduce uncertainty in system state and to reduce uncertainty in expected response to management actions. In the moose example (Fig. 3), the level of uncertainty associated with responses of this species to alternative forms of harvest regulations indicates that an adaptive management framework would likely be the most efficient approach to increase learning and, thus, better inform decision making.

Adaptive management contrasts outcomes from different actions and thus is only appropriate in settings with multiple pre-determined management actions; it is most effective in settings where managers face many of the uncertainties described in step 3—structural, environmental, partial controllability, etc. (Gregory et al. 2006; Walters 2007; Williams et al. 2009; Allen and Gunderson 2011). It is best suited to differentiating among broad categories of actions rather than among actions that differ in only small details (Moore et al. 2011), for which designed experiments work better.

Note that if structural and environmental uncertainty are the major concerns, monitoring under adaptive management should, ideally, lead to learning, allowing an eventual shift to effectiveness monitoring (as “which action under which condition” is resolved) and, perhaps eventually, just threshold monitoring (as structural uncertainty is adequately resolved).

Having identified the appropriate type of monitoring, it is time to move on to designing the technical details.

#### Step 6: translate the conceptual model from step 3 into quantitative form

The conceptual model and results chains formulated in steps 3–4 serve as the basis for developing a quantitative model or models (Irvine et al. 2015). The quantitative model(s), in turn, provides predictions regarding how the system will change in response to specific stressors or management actions. Model specification leads to consideration of the statistical methods that will be used to analyze the data and inform the design of the data collection (Reynolds 2012). Thinking through the models, analyses, and design consequences ahead of data collection speeds the rate of learning.

The degree of sophistication of the model(s) should be dictated by its intended uses and may require assistance from technical experts during development. Models should capture the key elements of a complex system or decision problem (Starfield 1997; Conroy and Peterson 2013). A quantitative model allows low-cost exploration of “what if” scenarios in which model inputs can be changed and model outputs examined. This is a powerful tool for designing monitoring but is rarely used in our experience.

##### Step 6a. What attributes are of interest and how should they be measured?

The objective hierarchy, initially sketched in step 2, and the results chains developed in step 4 can be used to explore and select the characteristics, or attributes, of the system that will be monitored. Biodiversity, abundance, survival, growth rate, habitat quality, and harvest rate are all system attributes that a monitoring program might seek to quantify. Returning to the moose example (Fig. 2), the fundamental objective can be stepped down to attributes that reflect success, such as productivity and population size. To help choose attributes, one can ask the question, “If I were fully successful in achieving my objectives, what would it look like? Conversely, if I failed to achieve my objectives, what would that look like?” Alternatively, when objectives lack specificity, one might ask, “In what ways do I expect the system to change from the way that it is currently?” Answering this question might lead to revised, more sharply defined objectives.

An ideal attribute responds directly to the possible actions (lagged and indirect effects are more difficult to interpret) and is not sensitive to other system components that are highly variable, out of management control, or difficult to measure. The ideal attribute should also be simple and easy to measure. Unfortunately, ideal attributes often do not exist and proxies are used instead; e.g., fish health may be of primary interest, but weight and length are more easily measured, and a condition index, weight divided by length, is calculated (see Olsen et al. (1999) for further discussion on this topic). It may take several iterations to get the fundamental and means objectives aligned with attributes that are feasible to measure, given program resources (Irvine et al. 2015). It is important to recognize when it is not feasible to measure the necessary attributes with the required precision given existing resources (Reynolds et al. 2011; Reynolds 2012); this is an especially important “reality check” once data become available.

We explicitly distinguish attributes from their physical measurement. When defining the measurement process (sometimes called the “response design” in the statistical literature) that will be employed, it is useful to consider three questions: How will the attribute be measured? What measurement scale is appropriate? What objects or individuals will be measured?

##### How will the attribute be measured?

Attributes that are commonly measured in natural resource contexts—e.g., body condition, habitat quality, biodiversity, abundance, and survival—can often be measured in many different ways. For example, plant abundance might be quantified by counting stems, visually estimating percent cover, or weighing harvested biomass and growth rate by recording change in height or diameter. As noted above, fish health could be measured as length-adjusted weight or, alternatively, by a visual inspection and classification as good, fair, or poor condition. In the moose example (Fig. 2), productivity is logically measured as the number of calves produced annually per cow, but the time post-calving when the count is taken also must be defined as part of how the attribute is measured.

##### What measurement scale is appropriate?

The scale of measurement determines the statistical methods that can be used to analyze monitoring data and runs from categorical through ordinal and interval to ratio [see Sparks-Jackson and Silverman (2010) for a detailed description of these measurement scales.] Measurements “higher” on the scale are more amenable to quantitative analyses, but often more prone to measurement errors and other problems. For example, in the field, it is generally easier, but less informative, to classify abundance as none-low-high (ordinal) than to count individuals (ratio).

##### What object will be measured?

The object, individual, or “thing” to be selected and measured must also be considered. In statistical terminology, this thing is the *sampling unit* (caution: do not confuse this use of “unit” with the *unit of measurement*, such as m, s, or °F.) In some cases, the sampling unit is a well-defined, natural object, such as an animal, tree, pond, nest, etc. More often, the sampling unit is arbitrarily defined, e.g., a transect, plot, core, net haul, etc. If sampling units do not have natural boundaries, one must choose the size, shape, depth, duration, or other relevant characteristics. There are a number of useful references that describe factors to consider when defining a sampling unit for measurement (Elzinga et al. 2001; Keeney and Gregory 2005).

Choice of measurement is critical, affecting the cost of the monitoring program, skills required of observers, the sampling designs one might employ, the analytical methods that are appropriate, and, ultimately, learning. In addition to considering the possible field and analytical methods available and their cost, it is worth thinking about three properties that will affect the usefulness of the measure.

First, how repeatable or variable is the measurement? Variability that results from the planned actions or other system components of interest is good, but uncontrolled variability and low repeatability will make learning difficult. For example, siphon pumps are used to catch aquatic micro zooplankton. Counting the numbers of each species requires considerable expertise; measurement errors are common. Moose may be counted via transects conducted by air; significant training is required to ensure repeatable counts among observers.

Second, could the measurement be biased? If so, can the bias be controlled or measured and adjusted for? One common form of bias results from imperfect detection, when organisms are missed by observers. If detection varies by habitat, condition, density, or observer, patterns in the data may reflect detection effects, which may obscure actual changes that monitoring is designed to track. Moose counted via aerial surveys are expected to be more detectable in open habitats compared with dense forests. Data collection can be designed to accommodate estimation of detection probabilities (MacKenzie et al. 2006).

Finally, is the measurement likely to produce a large number of zero values? Zeroes are common when measuring abundance in natural communities, e.g., secretive marsh birds. Zeros may represent a true absence or detection problems at low abundance (Martin et al. 2005). The second case is more problematic, but, in either case, a high prevalence of zeroes can complicate statistical analyses and data interpretation. Recognizing these complications early can lead to changes in sampling designs, analytical methods, or the selection of alternative attributes or measurements.

##### Step 6b. Modeling system change over time

Given the conceptual model formed in step 3, the anticipated actions from step 4, and some idea of the relevant attributes and measures, a quantitative model that describes both the natural system and the effect of management actions, if any, can be formulated. In general, it is better to start with a simple model and add complexity only as needed to resolve the problem or question rather than beginning with a complex model and trying to simplify (Starfield 1997). The initial model, based on the influence diagram (Fig. 3), should link key ecosystem drivers, anthropogenic drivers, or management decisions to desired outcomes. Various authors have addressed the problem of model formulation for fish and wildlife systems (Starfield et al. 1994; Hilborn and Mangel 1997). For the sake of brevity, we focus here on some relatively simple quantitative models with reference to the four types of monitoring identified previously (Table 1).

**Table 1:** Simple models for estimating attribute changes over time and in response to management

Model description	Model formulation
A. Simple linear trend: *β*_1_ > 0. Density is increasing *β*_1_ < 0. Density is decreasing	*y*_*t*_ = *β*_0_ + *β*_1_·*t* + *ε*_*t*_
B. Simple linear predictor	*y*_*t*_ = *β*_0_ + *β*_1_·*x*_*t*_ + *ε*_*t*_
C. Simple linear predictor with lag	*y*_*t*_ = *β*_0_ + *β*_1_·*x*_*t*_ + *β*_2_·*y*_*t*-1_ + *ε*_*t*_
D. Simple linear predictor with lag and step change due to action. *I*[*t* > 2004] = 1 if *t* ≥ 2005 and =0 for years prior to 2005	*y*_*t*_ = *β*_0_ + *β*_1_·*x*_*t*_ + *β*_2_·*y*_*t* − 1_ + *β*_3_·*I*[*t* > 2004] + *ε*_*t*_
E. Simple linear predictor with lag and linear response to management manipulation. *a*_*t*_ is the “off-limits” hunting acreage set aside in the management unit in year *t*.	*y*_*t*_ = *β*_0_ + *β*_1_·*x*_*t*_ + *β*_2_·*y*_*t* − 1_ + *β*_3_·*a*_*t*_ + *ε*_*t*_

For the moose management example, *y*_*t*_ denotes moose density in one management unit during year *t*, *x*_*t*_ denotes the number of hunting licenses issued in year *t*, and *ε*_*t*_ is the difference between the actual moose density and the density predicted by the model

##### Quantitative models when monitoring without or before management action

The primary role of a model in both status and trends and threshold monitoring is to summarize relationships among the major drivers of the system and the fundamental objectives, as represented by the attribute(s) of interest. These models may use time as the sole predictor of system response (Table 1(A)), may include covariate attributes that predict the attribute(s) of interest (Table 1(B)), or may include lagged variables (Table 1(C)).

Although the simple linear trend model provides a description of the past (Table 1(A)), it provides no insight into what caused the trend. If the unexplained changes in the response (*ε*_*t*_ in Table 1) are large relative to the trend, it will take a long time to detect the trend. An alternative model postulates that the response being measured is changing as a function of another attribute (Table 1(B), where the factor *x*_*t*_ for the moose example is a measure of hunting pressure). A more complicated model recognizes that 1 year’s value may well be related to that of previous years (Table 1(C)).

The models can become increasingly complex. For example, the response of multiple attributes could be modeled simultaneously, including relationships and interactions between them. Although statistical methods allow for such complex models of associations among system attributes, these models are limited in the insight that they provide into the causal relationships [though see Grace (2006) for an approach that accounts for our scientific understanding of direct and indirect relationships among attributes]. Another drawback of complex models is that the data requirements are usually higher than for simpler models; i.e., more attributes need to be measured.

When planning threshold monitoring, it is important to establish the threshold value or management trigger of the measured attribute in step 6, if not before. The basis for selecting the threshold should be documented, and the statistical methods that will be used to establish that the threshold has been crossed should be considered along with model formulation (Guttorp 2006).

##### Quantitative models when monitoring to evaluate management actions

Effectiveness monitoring and adaptive management focus on reducing the uncertainty surrounding system response to management actions. Unlike the quantitative models for monitoring without action, these models include a predictor variable that indicates when a new management action was implemented (e.g., Table 1(D) models a “step change” or constant shift in the system state as a result of action) and possibly reflects a measurement associated with the action (e.g., Table 1(E), where management action is quantified by the amount of acreage off-limits to hunting).

Quantitative models are central to the adaptive management framework, first encapsulating the system response expected as a result of potential management actions and then summarizing the observed response into rules guiding the next round of decisions (Kendall 2001; Lyons et al. 2008). These rules are often reflected in “model weights” assigned to each of a set of competing models describing system response. Weights are updated as monitoring data accumulate under different management actions, providing the quantitative learning process at the heart of the framework and thus reflecting the reduction in structural uncertainty.

#### Step 7: design the survey, the analytic approach, and the data management system

Having defined the required measurements and the quantitative model intended for synthesizing the data that are collected, it is time to design the details of the data collection, analysis, and data management components. These three components are very closely related, as the survey design will be informed by the intended data analyses and will, in turn, constrain any other supplemental analyses. For example, the best survey designs for assessing trends are usually quite different from the best survey designs for fitting a model to specify current relationships among a variety of attributes (McDonald 2012). Working out these technical details often entails collaboration with specialists in each component.

##### Step 7a. Design the survey

Survey design entails specifying where, when, and how to measure, given the available resources. This step will nearly always benefit from consulting a statistician or sampling design expert. Survey design is informed by a wealth of statistical research, and it is difficult for monitoring practitioners, who undertake design relatively infrequently, to gain or maintain this expertise. Many resources provide guidance on these topics (Elzinga et al. 2001; Thompson 2004; Schaeffer et al. 2011; Thompson et al. 2011; Reynolds 2012; Thompson 2012). The challenge for the practitioner is to know what guidance is best applied to their particular problem. A statistician familiar with natural resources problems and sampling design applications can guide the practitioner to the right resources, evaluate proposed sampling designs, and assist in planning the associated data analysis and reporting. Given the potential cost of any rigorous monitoring program (even one that involves only one field station), *this consultation should be considered essential, not a luxury*. The statistical consultation will be most efficient if those designing the monitoring have done their homework in steps 1–6. You may discover that what you want to learn cannot be obtained through monitoring, given time and cost constraints, and that it is more productive to focus your efforts elsewhere (Legg and Nagy 2006; Runge et al. 2011). This insight alone is worth all the effort devoted to following the road map in steps 1–6!

##### Sampling one time

Data are required to fit the quantitative model so it can be used to assist decision making. Commonly, the data come from observational studies that involve sampling. Statistical sampling allows for valid, unbiased inference from a set of observations (the *sample*) to the larger set from which they were selected (the *sample frame*), eliminating the need to conduct a complete census, which is usually infeasible. Statistical sampling methods provide measures of the uncertainty associated with the sample estimates (e.g., uncertainty of the estimated mean), allowing one to assess differences between the estimated characteristics and pre-determined reference values [see Reynolds (2012), for further discussion]. For example, is moose density, as estimated from a sample of aerial survey transects, above the threshold density required to allow a hunting season on the management unit?

Choosing the sample requires making a suite of decisions regarding (1) the individuals, objects, or locations being selected for measurement (i.e., the sampling units); (2) the population or area that we are selecting from and therefore able to make inferences about (i.e., the sample frame); (3) the population or area that we want to understand (i.e., the *target universe*); and (4) the rules or method for choosing sampling units. These decisions require careful consideration to avoid or clarify any potential sources of bias. Further, the decisions must be documented—both for future data collectors, so as to avoid introducing bias, and for future data users, so they can properly analyze the data (for further information, see the references listed in the first paragraph above) (“Step 7a. Design the survey” section).

Different choices among the elements 1–4 above result in tradeoffs between statistical precision and cost and incorporate different amounts of knowledge about the structure of the system under study. For example, an aerial survey of moose may stratify on distinct types of habitat (e.g., riparian corridors versus open tundra) to allow for more precise estimates than from a simple random sample, or logistical constraints associated with field access may make a simple random sample of vegetation communities prohibitively expensive and but a systematic sample more feasible (at the potential of some loss of precision). The number of possible methods and the importance of these tradeoffs make it best to develop the sample design through a collaborative team of experienced field technicians, statisticians, and managers. More complicated survey designs generally entail more complicated analyses and other potential constraints on broader use of the data (Reynolds 2012).

##### Sampling through time

Monitoring adds additional decisions about how frequently and for how long one should monitor, and how sample selection at one time relates to selection at another [the statistical literature refers to this as the “revisitation design”; see McDonald (2012) and Urquhart (2012)]. At one extreme is the decision to select survey locations once and revisit them on all future surveys (i.e., “repeated measures,” “longitudinal,” or “panel” design); this maximizes the ability to detect changes through time but limits coverage of the sample frame. At the other extreme, an entirely new set of locations is selected at each time point (“cross-sectional” design), which maximizes coverage of the sample frame through time but potentially at the cost of reducing the ability to detect changes in the response of interest (because of the added noise from changing locations). The opportunity to select new locations allows for the potential to broaden the total proportion of the sample frame that is measured, which may be a goal (often termed “coverage”). In between these two extremes are a range of approaches that provide different tradeoffs between the dual goals of coverage and detection of change, e.g., rotating panel designs. The ultimate design decision needs to consider project-specific factors, such as logistics (is it feasible to visit a new selection of locations each survey?), costs, effect of repeating data collection at a location (e.g., will repeated visits damage or otherwise change the features being measured?), and the complexity of the analyses that the design will dictate.

The repetition that comes from sampling through time raises the potential for problems to arise due to changes (sometimes subtle) in the sampling design elements. For example, if protocols are not specific about the timing of data collection, logistical or other pressures may arise that systematically shift the timing of data collection earlier or later in the season. If this changes the phenological state being sampled (e.g., growth stage), then the final monitoring time series will be confounded with changes in phenological state (see Reynolds (2012) for other examples).

##### Sample size determination

Determining an adequate sample size to accurately estimate key attributes is not a trivial problem. Sample size is a tradeoff between accurate estimation of key attributes and cost constraints (Legg and Nagy 2006). Ecosystem models can be complex, with attributes that can have a wide range of values and environmental variation that is difficult to predict. There are multiple sample size decisions that involve deciding how many samples to collect at a single location, multiple locations, and over time. In the moose example, decisions will need to be made about how to count the animals, when (in what seasons), the frequency of surveys (multiple times a year? every year? every third year?), and the number of sample units to measure during a single survey.

Computer simulation is the primary tool for calculating sample sizes; simple formulas generally do not exist (Smith et al. 2010; Thompson et al. 2011), another aspect of monitoring design best handled by a statistician or modeler. It is useful to collect pilot data to estimate sampling and temporal variation and test protocols and then use the estimates to derive sample sizes (Archaux 2008) when such information is unavailable from other projects or the literature. This differs from the “collect data” step in the road map, which refers to collection of project data during the implement and learn phase. In any event, once data are acquired, one should revisit the sample size analyses (see phase 4).

##### Step 7b. Design the analysis

Like the survey design, the data analysis approach reflects tradeoffs among information needs (step 2), feasible data collection designs, reporting deadlines, and technical skills of staff and should be guided by the quantitative models formulated in step 6. Because of the interdependence between survey design and analysis, these components must be thought through prior to data collection so as to ensure that (1) the analysis approach meets the program objectives and addresses the intended information needs and (2) is supported by the chosen survey design. Planning this ahead of data collection also allows for alignment of resources necessary to ensure that the analysis is conducted in a timely manner. One should avoid a long investment in collecting monitoring data only to discover years into it that the data do not support the proposed analysis nor answer the motivating question(s) (Legg and Nagy 2006).

There are potentially three stages of analysis, and fitting models is just part of one stage. The first stage is to summarize the most recently collected data using graphical (histograms, boxplots, and scatterplots) and numerical (averages, medians, percentages, standard deviations) methods. These should be reviewed with an eye toward data checking and exploratory data analysis to catch data transcription errors and other QA/QC issues.

Depending on the model to be fit and the fitting method, a potential second stage is to calculate the summary statistics associated with the selected attributes (Fig. 2), e.g., $\widehat{N}$, and its standard error and confidence interval, for estimating population size. The appropriate calculations will depend on the attribute and the survey design. For threshold monitoring, this is also when one assesses whether an attribute appears to have achieved the specified threshold value. These results provide information on progress toward the achieving the program’s objectives.

The final stage is to analyze the full data set (recent and historical data). This might include constructing simple time series plots, e.g., estimates of population abundance over time with associated confidence intervals. Models formulated in step 6 usually are fit using the entire data set, including data from past surveys. The models can be as simple as a linear model of the measurements (or their survey summary statistics, e.g., ${\widehat{N}}_{\mathrm{year}\ i}$) against time, e.g., a trend line (see model A in Table 1, as well as models B and C). Such time series plots and trend line models might suffice for status and trends monitoring (see step 5). For threshold monitoring, the analysis could involve a statistical test (which implicitly involves an underlying model) of a hypothesis that a threshold level has been reached, although confidence intervals constructed in the second analysis stage may suffice.

Effectiveness monitoring and *adaptive management* involve models that incorporate measures of management action(s) or their effects. These might be as simple as step change models [e.g., model D in Table 1 and various before-after and BACI analysis procedures (Smith 2002)] or more involved linear models (e.g., model E in Table 1), or they could be more complex. In this type of monitoring, analysis goals include estimating the current state of the system, assessing change in system state, and determining whether a management action had the predicted effect. These last two goals require data from at least two time periods—before and after the action.

Monitoring data for adaptive management will also be analyzed to assess the predictive performance of the quantitative models used in developing the most recent management guidance. For each of the attributes of interest, how close were the predictions of current system state (made prior to monitoring data collection) to the actual monitoring observations? If competing quantitative models had been fit prior to the most recent survey, then usually, the models will be refit using the newly expanded data set as well as calculating a new estimate of the relative strength of evidence for each model, e.g., update the model weights (Kendall 2001; Kendall and Moore 2012). This information would be used in step 10 to guide future management.

We strongly encourage development and use of mechanistic models in analyzing monitoring data rather than relying on “simple” empirical summaries such as simple linear regression models (Hilborn and Mangel 1997; Bolker 2008). Observing or quantifying empirical trends is only a first step in understanding the system being monitored. Intervention in the form of management requires understanding how the system operates, not just knowing that it has changed (Irvine et al. 2015).

##### Step 7c. Design the data management system

Data stewardship should begin in advance of actual data collection and should address both the immediate data analysis needs and long-term archiving of data for future uses, including those not currently anticipated (Reynolds et al. 2014). A common pitfall of monitoring programs is to devote most of the resources to data collection and very little to documenting, managing, and using the data to inform decision making. Yet, error-free, well-documented data are the basis for defensible decision making. Well-designed data management systems greatly improve efficiency of data collection, as well as the ease and speed of planned analyses and reporting, and thus the effectiveness of monitoring for informing management decisions (Lane et al. 2004; Reynolds et al. 2014). However, comprehensive attention to data management is not standard practice in the conservation science community. Studies suggest that 80 % of the data used as the basis for peer-reviewed science publications becomes irretrievably lost within 20 years (Gibney and Van Noorden 2013; Vines et al. 2014) and the rate of loss is expected to be even higher for “unpublished” data commonly generated by natural resource management agencies. A broad estimate of ∼30 % of a monitoring program’s total budget (time and staff) should be allocated to data quality control and management (Fancy et al. 2009); commonly 10–15 % of total budget is devoted to monitoring.

Attention to long-term curation is especially important given the lifespan of many monitoring programs and the potential value of these data for currently unforeseen future applications—by the monitoring program, its stakeholders, or other users. Curation includes making data discoverable by others (and, thus, increasing the return on the data collection investment) and instituting guidelines and systems for data sharing. Data and metadata must remain linked together and be thorough enough to allow someone unfamiliar with the project to interpret the data. For “flat files” of data stored in electronic spreadsheets, a separate sheet for metadata is convenient. However, centralized, web-based data management systems are essential for collaborative monitoring projects where many co-operators collect the same data; these systems require significant advanced planning and budgeting to ensure that required technical capacities are engaged (Hunt et al. 2015; Hunt et al. in press). Such online systems allow for greatly improved data discovery and delivery, achieving greater value from the data investment (Reynolds et al. 2014).

As with statistical design and analysis, it is beyond the scope of this paper to detail all the necessary steps and technical considerations in designing and implementing data management for a monitoring program. In short, data documentation, including accurate and complete project metadata (who, where, what, how, when) and data metadata (what is encoded in each data field—the data dictionary), are essential. If management actions are implemented, the database must capture the timing and implementation conditions, as well as any problems that arose (e.g., partial implementation). Similarly, the database must capture any changes in the survey design or implementation. Failing to record this information may make it impossible to discern accurately the impact of the action. Maintaining the project record, started in phase 1 and continuing as the monitoring design and implementation proceeds, is essential.

##### Step 7d. Write protocols

Another common pitfall of monitoring programs is lack of well-written protocols. The protocol summarizes all the decisions made as one progresses through the road map, including all survey design, data collection, data management, analysis, and reporting plans, as well as hiring considerations and training (Oakley et al. 2003; US Fish and Wildlife Service 2013). In addition, the protocol includes specific instructions for all procedures (standard operating procedures). The protocol must contain clear, detailed, and unambiguous procedures so that those unfamiliar with the project can learn to implement it. This documentation is essential for the analysis of the data, as well as for ensuring that the data collection is replicable and defensible and avoids confounding. Of equal importance, the protocol describes how the monitoring information will be used, to either track resource status or inform future management. Most new protocols require at least two sampling seasons to pilot-test for feasibility, replicability, logistics, cost, and training requirements.

### Phase 3. Implement and learn

#### Step 8: collect and manage data

With a written protocol as a guide, one is ready to implement the survey (at last!). As with the other steps, the details of the elements involved in step 8—including safety measures, training, permitting, and logistics, etc.—require time and resources to plan and will vary widely in their details. Ideally, at least one cycle of monitoring is done prior to implementing any management. However, in many settings, management is underway prior to monitoring.

#### Step 9: analyze data and report results

The purpose of monitoring is to generate useful information to inform the motivating question(s) (step 1). Completing steps 1–8 should ensure that the necessary resources and systems are in place for timely analysis and summarization of the new information and its communication to the decision makers identified in step 1. Delayed analysis and reporting not only reduce the information’s value but also delay potential identification of problems in the alignment of the survey design, data collection, planned analyses, and monitoring objectives—issues that should be detected and resolved quickly. *Problems with data quality or interpretation often only come to light when the data are summarized*, *graphed, and analyzed.*

##### Data analysis and modeling

Analyses should follow the plan developed in step 7. They should provide an assessment of how close the current state of the system is to the state implied by the fundamental objective. For example, if the fundamental objective is to restore an endangered species, then data analysis should, at a minimum, describe the current state of the species and, for example, calculate an estimate of abundance and its standard error.

Analyses should include adequate basic exploratory data analysis (EDA) and model diagnostics. These generally focus on statistical graphics to screen for “odd” data that may signal errors in collection or management of the data and to assess the adequacy of the quantitative models (step 6) used. There are a number of cautions to keep in mind in when engaging in EDA, however. For example, data dredging can result in apparent “statistically significant” results arising from randomly generated patterns (Anderson et al. 2001a; Anderson et al. 2001b). Do not immediately assume that the initial model(s) adequately summarizes the observed relationships. This is especially important if knowledge of system behavior is limited and/or the system attributes are relatively variable. Not all quantitative models readily lend themselves to “off-the-shelf” diagnostics (see, for example, Harrell (2002); Ryan (2008), and Fox (2011) for tools for such assessments and diagnostics).

##### Reporting and feedback to management

Timely reporting means that decision making improves over time, managers are able to defend their management decisions, and the survey is viewed as too important to cut from the budget. In adaptive management, reports must be delivered prior to planning the next management action. The presentation of results should be focused explicitly on key information needs defined in step 1, as well as caveats and insights from the analysis. Information will likely need to be presented in multiple ways to address the needs of different stakeholders. Decision makers will want the monitoring information translated into user-friendly decision support tools. The science community expects peer-reviewed journal papers; agency leadership will also want a brief synopsis of findings or outcomes. Databases can be programmed to generate drafts of the needed reports as soon as the monitoring data are entered and proofed (Hunt et al. 2015).

#### Step 10: update models, assess, or plan and implement actions, when relevant

Monitoring data are used to update system models under all forms of monitoring. In addition, for monitoring associated with management actions, the updated models inform the next cycle of management. The primary purpose of status and trends monitoring is to improve system understanding; therefore, the system model is refined by integrating the new observations into the quantitative model developed in step 6. The updated system model may stimulate interest or concern from policy makers, especially if the system seems to be heading in an undesirable direction. Policy or management planning may ensue. For monitoring to detect a threshold condition, management actions are anticipated and defined at the outset. When an attribute is estimated to pass a specified threshold (step 9), management actions are considered.

For effectiveness monitoring, the state of the system is observed before and after an action has occurred, leading to learning. If the outcome of the action is unsatisfactory from a management standpoint, this may prompt a return to phase 1 (frame the problem) to consider multiple actions, perhaps under a formal adaptive management framework. Under adaptive management, the models and management actions are fully specified during steps 1–7. Models are updated with the monitoring data in step 9 on a defined schedule (often annually). The model with the strongest weight of evidence provides guidance regarding the most effective management action to undertake in the future. The iterative process of updating the system model with new information derived from the monitoring data is ongoing (steps 8–10) until system understanding is deemed adequate for clearly distinguishing among the potential management actions.

### Phase 4. Learn and revise

Few monitoring programs last indefinitely. Perhaps, the problem or question that prompted the project is resolved or has receded in importance. More commonly, other emerging problems will be judged more pressing and monitoring effort will be redirected. Changes in the problem itself or advances in management or monitoring tools may require revisiting the purpose and design of the survey and, potentially, initiating changes. During the learn and revise phase, the entire survey is reviewed using the same process that was used to plan a new one.

**Fig. 5:**
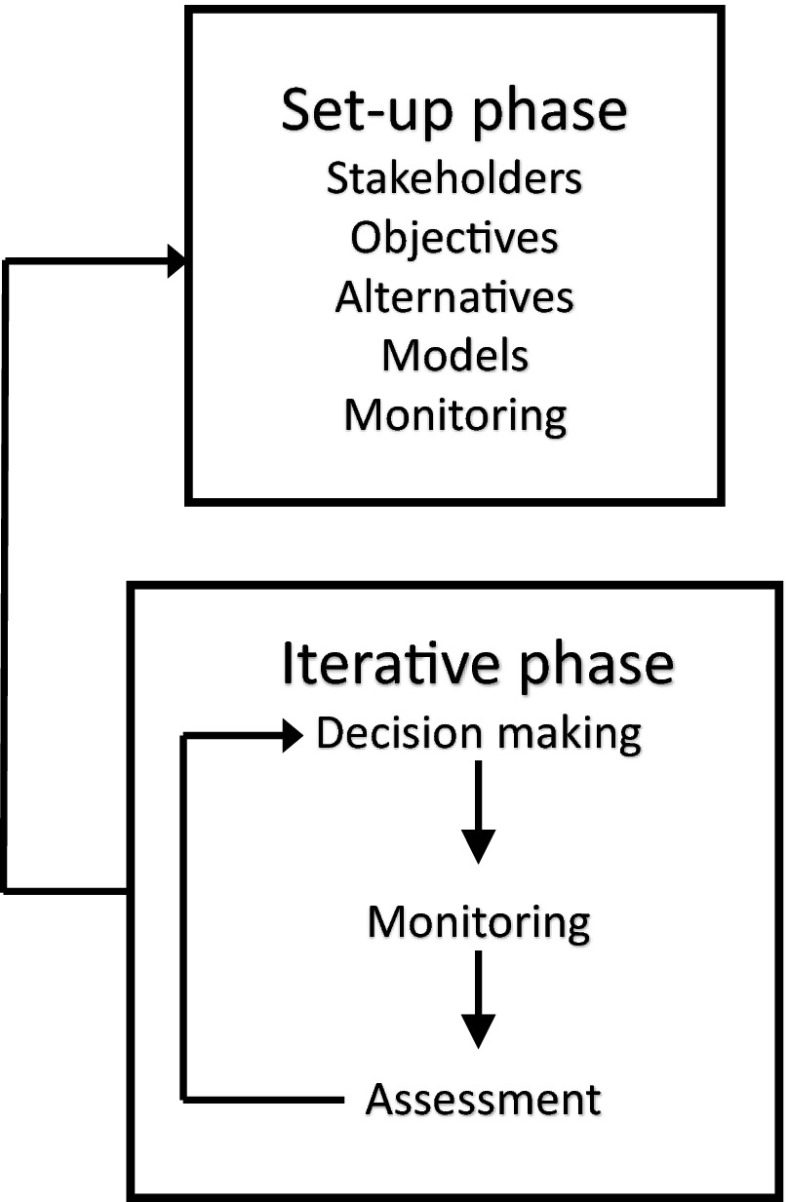
The two phases of learning distinguished in adaptive management (Williams et al. 2009), shown above, also occur in the road map. Technical learning involves an iterative sequence of decision making, monitoring, and assessment (road map steps 8–10). Process and institutional learning involve periodic reconsideration of the set-up elements (road map phase 4—learn and revise)

If all the steps were not considered or discussed when the survey was planned, now is the time to do so. Reviewing an existing monitoring program with the road map as a guide will result in improvements to the survey. Or, a review may lead to a decision to end a survey that has achieved its purpose or has failed to produce useful information (Possingham et al. 2012). Adaptive management has a built-in mechanism (double-loop learning) for revisiting the structuring phase and revising or ending the survey (Williams et al. 2009; Williams et al. 2012); the learn and revise phase represents double-loop learning under adaptive management (Fig. 5) but is equally applicable to all types of monitoring. Under threshold monitoring, if years have elapsed since the monitoring was initiated, new management options may be available. If system understanding has advanced in the intervening time, a revision of the threshold condition may be advised.

The need for “pause and reflection” is especially important for status and trends monitoring (Renner et al. 2011; Irvine et al. 2015). Because this type of monitoring tends to have less clearly defined objectives, it is essential to regularly schedule analyses to synthesize the results and check that the information produced remains relevant. Use what has been learned about the system, especially the estimates of variation, to check whether the sampling design and effort levels are adequate (Reynolds et al. 2011) or can be made more efficient (Renner et al. 2011).

Even if the motivating problem or decision remains a priority, the goal is for system learning to reach a level where formerly major sources of uncertainty are reduced enough to change the type of information needs motivating the monitoring. For example, such a change may allow one to shift from, say, effectiveness monitoring to threshold monitoring or monitoring under adaptive management to effectiveness monitoring. Thus, an operational goal of all monitoring should be to continually improve not only in terms of effectiveness (methods, sample sizes, efficiencies) but also in terms of the motivating decision context and information needs.

## Summary

In our experience, monitoring programs that succeed have a clear purpose, strong leadership, and accurate documentation and emphasize team work and collaborative learning. The road map provides a big-picture perspective of a process that can be applied by individuals or teams engaged in planning new monitoring, as well as those reviewing the relevance and scientific rigor of ongoing monitoring. Specifically, it emphasizes the many initial planning steps required to produce useful information to meet the needs of policy or decision makers. It is a unified approach that can be applied to any type of monitoring, including status and trends, threshold, effectiveness, and formal adaptive management. It can be used for programs of any size and can be applied by staff with a wide range of technical and scientific skills.

Many people who design monitoring programs begin with step 8 (collect data), failing to explicitly clarify the problem, objectives, and management alternatives; build a conceptual model; and identify the relevant system features and decision maker information needs. They have not thought carefully about what success looks like, what attributes to monitor, how they will be measured, or considered the data analysis or any quantitative models. *These oversights are why monitoring programs so often fail to deliver as expected.*

It is easy to get lost in the details of designing monitoring. The road map is a guide to the overall process, a reminder to keep the big picture in mind, even when dealing with technical details. It provides a set of benchmarks (steps) that can be used during the design phase to keep projects on track, schedule statistical consultants, prepare budgets, and plan program evaluations for existing monitoring projects. It does not address all the underlying technical details of each step; specific guidance can be found in the appropriate literature for each component or task. The road map helps ensure the value of monitoring information, now and in the future.

## Appendix. Documenting monitoring in a project record

The project record is an administrative record of a monitoring project or program. It is a “living” document that is updated over time as the project evolves. It is best to begin documentation as soon as phase 1 (frame the problem) is completed and before moving on to phase 2—design. Comprehensive documentation of a monitoring program will support writing the monitoring protocol, summary reports, and journal papers. The project record is also essential for understanding the rationale for all aspects of the monitoring program, designing appropriate data analyses, interpreting monitoring results, and providing key historical information for program reviews and evaluations. Any changes to the monitoring program should be documented in the project record, along with the rationale for those changes. The project record should be archived with all key project documents, including the protocols and data sets. The outline that follows is a suggested format that can be modified to fit the needs of each project.

1. Introduction
  - Brief overview of the project, the problem, scope, and history
  - Links to the administrative history and evolution of the project (5a, below)
2. What’s the problem?
  - Problem statement
    (i) Decision maker(s)
    (ii) Types of decisions
    (iii) Geographic and temporal scope
    (iv) Legal/regulatory context and constraints
  - Objective hierarchy
    (i) Fundamental objective(s)
    (ii) Means objectives
    (iii) Attributes
    (iv) Measures
    (v) Threshold values, if any
  - Conceptual model of the system (influence diagram)
    (i) Fundamental objective
    (ii) Factors (system drivers)
    (iii) Management /policy decisions
    (iv) Summary of the literature, as the basis for the conceptual model
  - Management/policy decisions that will be informed by the monitoring (if any)
3. Monitoring design
  - Rationale for undertaking monitoring.
    (i) Alternatives considered
    (ii) Cost-benefit analysis
  - Type of monitoring selected and rationale [note: the details of the following documentation will differ, depending upon the type of monitoring selected]
  - Quantitative model(s)
    (i) Specify competing models and associated predictions
      1. Fully specified, SMART objectives
      2. Attributes
      3. Covariates
  - Data analysis plan
    (i) Derived from models above
    (ii) How will the analysis address the problem defined above (2)?
    (iii) Reporting schedule
  - Data management plan
    (i) Data sharing agreements
    (ii) Data archiving
    (iii) Requirements of the data management system
  - Protocol
    (i) Links to approved protocol documents
      1. Requirements will vary among agencies
      2. Will incorporate some information from the project record, with a focus on procedures necessary to produce and maintain a quality data set.
      3. Technical details (sampling design, measurements, observations)
      4. Administrative details (who, what, where, when)
        - Budget
        - Staffing
        - Training
        - Permits, legal documents
4. Implement and learn
  - Documentation of management actions/decisions, if any [note: this should also be in the database]
  - Updated models, based on learning from monitoring [note: under adaptive management, models are updated as monitoring data are entered and proofed]
  - Annual summary of monitoring schedule and activities, problems encountered, management recommendations, if any
5. Learn and revise
  - Administrative history of the project
    (i) Survey coordinators/principal investigators (science team)
    (ii) Cooperators (those participating in the monitoring), if more than one station or agency
    (iii) History of meetings, workshops, and conference calls
      1. Dates
      2. Outcomes/decisions
    (iv) Project evolution over time
      1. Budget history
      2. Changes to any aspect of monitoring design, rationale, and dates implemented.
  - List of reports, journal papers, presentations, workshops, products and links to archives.
  - Summary of how program products have supported management/policy decisions or helped to resolve the problem (2).
  - Summary of dates, outcomes, and recommendations from program reviews
  - Links to updated models, based on best available information (see 4b above)
  - Summary of dates and changes to monitoring program administration or monitoring design, with rationale.
    (i) Links to revised protocol documents
  - Implications for future data analysis and reporting. How will changes to the monitoring program affect future interpretation of the data?
  - If program will sunset, document rationale
